# Supplementary material for: A deleterious Nav1.1 mutation selectively impairs telencephalic inhibitory neurons derived from Dravet Syndrome patients
Source: eLife. 2016 Jul 26;5:e13073. doi: 10.7554/eLife.13073 (PMC4961470; doi:10.7554/eLife.13073)
Supplement: Supplementary file 2. — DOI: http://dx.doi.org/10.7554/eLife.13073.036 [file elife-13073-supp2.docx]

Supplementary file 2. Pluripotent stem cell lines characterized by electrophysiology

| Experimental  settings  Cell line IDs  Experimental  Readouts | **Neuronal intrinsic properties** | | **shRNA-mediated**  **Na_V_1.1 knockdown** | | **cDNA-mediated**  **Na_V_1.1 rescue** |
| --- | --- | --- | --- | --- | --- |
|  | Excitatory neurons | Inhibitory neurons | Excitatory neurons | Inhibitory neurons | Inhibitory neurons |
| I_Na_:  Voltage- dependent activation | Control-ESC-H9  Control-iPSC-8858-C  Control-iPSC-6593-8  Dravet-iPSC-2105-4  Dravet-iPSC-2105-12  Dravet-iPSC-6358-2  Dravet-iPSC-6358-3 | Control-ESC-H9  Control-iPSC-8858-C  Control-iPSC-6593-7  Control-iPSC-6593-8  Control-iPSC-NH2-6  Dravet-iPSC-2105-4  Dravet-iPSC-2105-12  Dravet-iPSC-6358-2  Dravet-iPSC-6358-3 | Control-ESC-H9 | Control-ESC-H9 | Dravet-iPSC-6358-2 |
| I_Na_:  Steady-state inactivation | Control-ESC-H9  Control-iPSC-8858-C  Control-iPSC-6593-8  Dravet-iPSC-2105-4  Dravet-iPSC-2105-12  Dravet-iPSC-6358-2  Dravet-iPSC-6358-3 | Control-ESC-H9  Control-iPSC-8858-C  Control-iPSC-6593-7  Control-iPSC-6593-8  Control-iPSC-NH2-6  Dravet-iPSC-2105-4  Dravet-iPSC-2105-12  Dravet-iPSC-6358-2  Dravet-iPSC-6358-3 |  |  |  |
| Action potential generation: maximal output and dynamic output | Control-ESC-H9  Control-iPSC-8858-C  Control-iPSC-6593-8  Dravet-iPSC-2105-4  Dravet-iPSC-2105-12  Dravet-iPSC-6358-2  Dravet-iPSC-6358-3 | Control-ESC-H9  Control-iPSC-8858-C  Control-iPSC-6593-7  Control-iPSC-6593-8  Control-iPSC-NH2-6  Dravet-iPSC-2105-4  Dravet-iPSC-2105-12  Dravet-iPSC-6358-2  Dravet-iPSC-6358-3 |  | Control-ESC-H9 | Dravet-iPSC-6358-2 |
| Time-dependent maturation of action potential patterns |  | Control-iPSC-8858-3  Control-iPSC-6593-7 |  |  |  |
| Properties of human neurons engrafted to rat hippocampal organotypic slices |  | Control-ESC-H9  Control-iPSC-6593-7  Control-iPSC-8858-C |  |  |  |

Notes: Control-iPSC-8858-C and Control-iPSC-8858-3 are two iPSC lines generated from two reprograming experiments

for the same control subject 8858. 8858-3 and another control iPSC line 8402-2 WT9 were included at later stages of this

study, and they were only used in experiments to further validate the neural differentiation protocol.
